# CMTM6 and CMTM4 as two novel regulators of PD-L1 modulate the tumor microenvironment

**DOI:** 10.3389/fimmu.2022.971428

**Published:** 2022-07-25

**Authors:** Tong Zhang, Haixiang Yu, Xiangpeng Dai, Xiaoling Zhang

**Affiliations:** ^1^ Key Laboratory of Organ Regeneration and Transplantation of Ministry of Education, First Hospital, Jilin University, Changchun, China; ^2^ National-Local Joint Engineering Laboratory of Animal Models for Human Disease, First Hospital, Jilin University, Changchun, China; ^3^ Department of Thoracic Surgery, China-Japan Union Hospital of Jilin University, Changchun, China

**Keywords:** CMTM6, CMTM4, TME, PD-L1, M2 macrophage, MDSC, Treg, CTL

## Abstract

The tumor microenvironment (TME) plays crucial roles in regulating tumor occurrence, progress, metastasis and drug resistance. However, it remains largely elusive how the components of TME are regulated to govern its functions in tumor biology. Here, we discussed how the two novel functional proteins, chemokine-like factor (CKLF)-like MARVEL transmembrane domain-containing 6 (CMTM6) and CMTM4, which involved in the post-translational regulation of PD-L1, modulate the TME functions. The roles of CMTM6 and CMTM4 in regulating TME components, including immune cells and tumor cells themselves were discussed in this review. The potential clinical applications of CMTM6 and CMTM4 as biomarkers to predict therapy efficacy and as new or combined immunotherapy targets are also highlighted. Finally, the current hot topics for the biological function of CMTM6/4 and several significant research directions for CMTM6/4 are also briefly summarized in the review.

## Introduction

CMTM is a family of proteins linking transmembrane 4 superfamily (TM4SF) and chemokines, containing a MAL and related proteins for vesical trafficking and membrane link (MARVEL) domain (1). This family is encoded by 9 genes including CKLF and CKLFSF1-8 in humans (1, 2). CMTM genes are mainly distributed on chromosomes 3, 14 and 16 and included two distinct gene clusters (1). The roles of the CMTM family have been reported in many previous studies, including involvement in occurrence of tumors and regulation of the immune system and the male reproductive system (3–11). For example, CMTM1, CMTM2 and CMTM3, which are highly expressed in testis, may play a significant role in spermatogenesis (7–9). In addition, CMTM3 can regulate angiogenesis (5). CMTM5 and CMTM7 demonstrate tumor suppressor activities by inhibiting the proliferation of tumor cells (3, 10). CMTM7 has also been reported to be involved in B cell receptor (BCR) signaling (6). CMTM8 has the function of inducing apoptosis and regulating epidermal growth factor receptor endocytosis (11).

In 2017, two studies simultaneously reported in Nature revealed that CMTM6 and CMTM4 are key proteins that regulate the stability of PD-L1 (12, 13). They found that CMTM6 and CMTM4, but not other CMTM family members, enhanced the expression of both inducible and constitutive PD-L1 at the cell membrane *via* protecting PD-L1 from 26s protease or lysosome-mediated degradation. In the absence of CMTM6, CMTM4 will alternatively exert the important function in regulating the expression of PD-L1 (13). PD-L1, encoded by the CD274 gene, is mainly expressed on the cell membrane surface of tumor cells and some immune cells including macrophages and dendritic cells(DCs) (14). PD-L1 binds to its receptor and inhibits the function of many immune cells, especially CD8^+^T cells (15, 16). As an immunosuppressive factor, PD-L1 is frequently upregulated in TME and thereby inhibiting the function of cytotoxic T lymphocytes (CTLs) and promoting the development of tumor (17)

CMTM6 has been shown to have oncogenic properties and be highly expressed in various tumors such as head and neck squamous cell carcinoma (HNSCC) (18), glioma (19, 20), colorectal cancer (21), ovarian cancer (22), oral squamous cell carcinoma (OSCC) (23, 24), hepatocellular carcinoma (HCC) (25, 26), and renal cancer (27) (**Table 1**). The high expression of CMTM6 is closely correlated with poor prognosis in different tumors (18–20, 24, 25, 27, 29, 32–37, 39) (**Table 1**). In addition to directly affect tumor proliferation, the tumor-promoting effect of CMTM6 may also be accomplished by indirectly regulating TME (18, 40, 41). CMTM4 and CMTM6 share 55% sequence similarity, and the former is the most conserved member of the family. CMTM4 expression is upregulated in HNSCC patients and correlated with worse prognosis (42). Therefore, CMTM6 and CMTM4 are potential biomarkers for predicting immunotherapy efficacy (28, 30, 31, 38, 43) (**Table 1**).

**Table 1:** Correlation of CMTM6 expression in different tumors with clinical prognosis and immune molecules in TME.

Reported tumor types (CMTM6)	Expression of CMTM6		Prognostic or clinical value	Related immune molecules in TME	References
HNSCC		↑	Poor prognosis	PD-L1, LAG-3, TIM-3, VISTA, B7-H4, B7-H3, CD4,CD8	(18)
OSCC		↑	Poor prognosis	N/A	(24)
		↑	N/A	PD-L1	(23)
Ovarian cancer		↑	Better prognosis	CD4, CD8	(22)
Gliomas		↑	Poor prognosis	PD-L1, PD-L2, PD-1, CD80, TIM-3	(20)
		↑	Poor prognosis	N/A	(19)
Colorectal cancer		↑	Favorable prognosis	PD-L1, CD4, CD8	(21)
		N/A	Biomarker for predicting PD-1/PD-L1 inhibitors	PD-L1, CD4, CD8, CD68, CD163	(28)
HCC		↑	Biomarker to predict the recurrence risk	CD8, PD-L1, PD-L2, B7- H3, and B7- H4	(26)
		↑	Poor prognosis	PD-L1	(25)
		N/A	Poor prognosis	PD-L1	(29)
Lung cancer		N/A	Independent predictor for PD-1 inhibitors	PD-L1	(30)
Renal cancer		↑	Worse prognosis	CD4, CD8, CD11b, CD68, PD-L1	(27)
		N/A	Biomarker for immunotherapy	PD-L1, CD3	(31)
Breast cancer		N/A	Higher risk for disease progression	PD-L1	(32)
Gastric cancer		↑	Shorter overall survival; enhances the prediction value of PD-L1	PD-L1	(33)
		N/A	Poor prognosis	PD-L1	(34–36)
Sarcoma		N/A	Worse prognosis	PD-L1	(37)
Melanoma		N/A	Potential predictive factor for ICI	PD-L1, CD3, CD20, CD68	(38)
Pancreatic cancer		N/A	Shorter overall survival	PD-L1	(39)

HNSCC, Head and neck squamous cell carcinoma; OSCC, Oral squamous cell carcinoma; HCC, Hepatocellular carcinoma; ICI, immune checkpoint inhibitors; N/A, not applicable. The "↑" means the expression of CMTM6 in tumor tissues is higher than that in non-tumor tissues.

TME is the ecosystem which includes the extracellular matrix, immune cells, blood vessels, tumor cells and other cells. The tumor and its surrounding TME interact with and influence each other (44). Firstly, TME remarkably influences cancer development through affecting metabolic, epigenetic, immune or other microenvironments (45). Secondy, tumor cells can shape and train the TME and ultimately help their survival and migration in an organism (44). Moreover, immune effector cells in TME are comprehensively reprogrammed as accomplices of tumor cells, which could protect cancer from immune destruction, subsequently evade immune surveillance and ultimately promote tumor progression and metastasis (17, 46–51). Accumulating evidences showed that TME might be a main obstacle for effective cancer immunotherapy (52, 53) and confers resistance to the immunotherapies (54). The in-depth understanding of the effect of tumor microenvironment on immunotherapy is necessary for developing novel immunotherapy strategies.

The microenvironment properties are important for TME in regulating the tumor occurrence and development. Stromal cells including vascular endothelial cells, fibroblasts and pericytes are the main components of TME (55–58). The immune effector cells and immune suppressor cells are the two populations of the immune cells in TME. The immune effector cells mainly include CD4^+^ T cells, CD8^+^ cytotoxic T cells and NK cells. The immunosuppressive cells mainly include Treg, Breg, M2-like macrophages and myeloid-derived suppressor cells (MDSCs) (59–63). Immunosuppressive cells can inhibit T cell- and NK cell-mediated tumor killing through multiple mechanisms and lead to immunotherapy resistance (64–66). In addition, cytokines, chemokines, growth factors and exosomes are also important components of the TME.

Recent studies have revealed potential links between CMTM6/4 and immune cells in TME. Not only CMTM6/4 could inhibit the activity of cytotoxic T cells by stabilizing PD-L1, but also CMTM6 can promote the polarization of M2 macrophages through exosome shuttling. Furthermore, CMTM6 and CMTM4 influence the functions of multiple important components of the TME. Here, we briefly summarized and discussed the latest progresses regarding the roles of CMTM6 and CMTM4 in regulating TME and its components, thus highlighted their roles in shaping the immune tolerance state.

## The expression of CMTM6/4 is associated with the immune-related signatures in TME

In addition to tumor cells, CMTM6 and CMTM4 are also expressed on antigen-presenting cells including macrophages and DCs (28, 41, 67). CMTM6 is associated with immune-related molecules in TME (**Table 1**). CMTM6 correlates with immune-associated pathway, infiltration of immune cells and the expression of most genes related to immune response in TME. The potential relationship between CMTM6 and inflammatory or immune response was explored in tumors such as gliomas (20), lung cancer (41, 68, 69), and ovarian cancer (22) by using functional annotation enrichment analysis, gene set enrichment analysis (GSEA), gene set variation analysis (GSVA), and other analytical methods. All the results indicated that CMTM6 might be crucial in modulating the TME.

Currently, most results indicated that CMTM6 helps to establish an immunosuppressive microenvironment in many tumors, such as gliomas, renal carcinomas and colorectal cancer (CRC) (20, 27, 28). Guan and colleagues demonstrated that CMTM6 modulated T lymphocyte-mediated antitumor immunity in gliomas (20). Gene ontology analysis results reveal that CMTM6 can influence the inflammatory activation and immune response of glioma (20). The GSVA results showed that CMTM6 could promote Treg differentiation and induce T cell tolerance. Wu et al. found that CMTM6 was negatively correlated with the infiltration of CD8^+^ T cells and positively correlated with the infiltration of M2 macrophages and CD4^+^ T cells (28). By establishing models of BALB/c mice implanted with CMTM6 knockdown Renca cells Wang et al. concluded that CMTM6 expression was negatively correlated with the intratumoral infiltration of CD4^+^ and CD8^+^ T cells, but positively correlated with MDSC and macrophages (27). Zhou et al. found that expression level of CMTM4 mRNA was negatively correlated with the infiltration of cytotoxic cells, DCs, and CD8^+^ T cells in HCC based on the TCGA database (70). Their results indicated that CMTM4 may play a significant role in TME and correlate with the infiltration of immune cells in tumor tissues (70). Altogether, the above results elucidated CMTM6 and CMTM4 play crucial roles in regulating the function of related immune cells and tend to participate in maintaining the immunosuppressive state of TME.

### CMTM6 regulates the functions of M2 macrophages in TME

As important components of TME, tumor-associated macrophages (TAMs) can be simply divided into two categories according to their functions: M1 and M2 macrophages. In contrast to the function of M1 macrophages, M2 macrophages are functionally characterized by immunosuppression and promotion of angiogenesis (71). Cytokines derived from tumors and tumor-associated immune cells educate macrophages in the TME to differentiate toward M2 macrophages (72). M2 macrophages in turn inhibits the activity of T cells and NK cells through immunosuppressive metabolites and cytokines (71, 73). M2 macrophages facilitate immune evasion of tumor cells by inducing the expression of CD47 and PD-L1 in various cancers including pancreatic cancer and OSCC (74–76).

**Figure 1:**
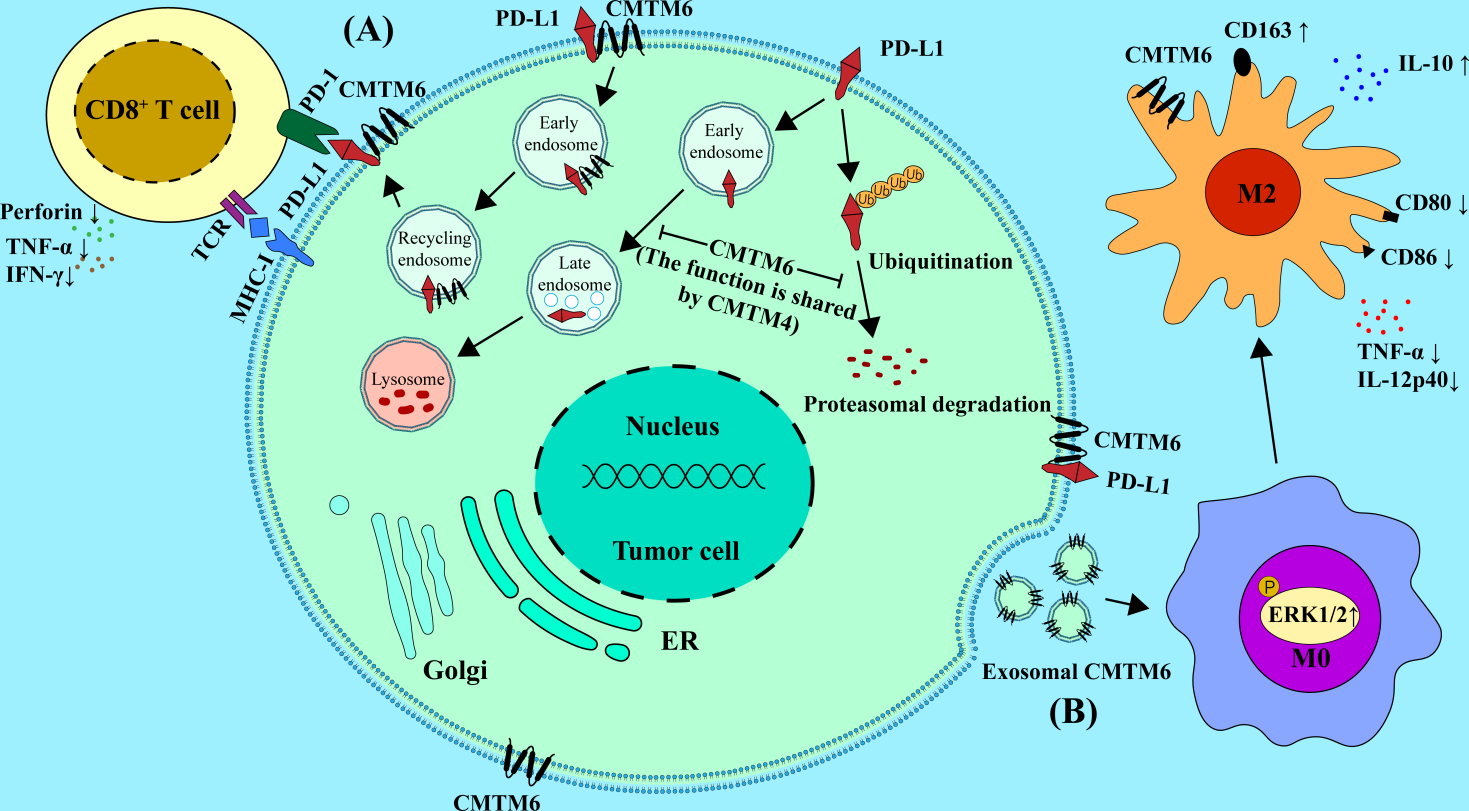
Mechanisms of CMTM6 and CMTM4 in regulating the function of the immune cells in TME. **(A)** CMTM6 protein can stabilize the PD-L1 protein on tumor cell, and this function is shared by CMTM4. CMTM6 co-localizes with PD-L1 at the plasma membrane and in recycling endosomes, where it inhibits PD-L1 being targeted for lysosome-mediated degradation. Moreover, CMTM6 inhibits the ubiquitination of PD-L1. CMTM6/4 enhancedPD-L1-mediated T cell suppression through the above pathways, as indicated by decreased secretion of perforin, TNF-α and IFN-γ.**(B)** CMTM6 promotes M2 macrophage polarization through exosome shuttling by activating ERK1/2 signaling in M0, whereas it inhibits M1 macrophage polarization. It is mainly manifested by the up-regulation of M2-specific markers such as CD163 and IL-10 and the down-regulation of M1-specific markers such as CD80, CD86, TNF-a and IL-12p40.

Recent studies showed that CMTM6 was related to M2 macrophage polarization. In addition, the infiltration proportion of M2 macrophages in tumor tissues was positively correlated with the expression of CMTM6 (68). A recent study revealed a new mechanism by which tumor cells interact with immune cells. The results demonstrated that the exosomal CMTM6 from OSCC cells could induce the polarization of M2-like macrophages in the TME (77). Moreover, in OSCC patients, a positive correlation was found between CD163+ macrophage infiltration and CMTM6 expression. Furthermore, by using a transwell system of tumor cells co-cultured with the PMA-differentiated human THP-1 monocytes (M0 macrophages), they found CMTM6 in tumor cells was negatively correlated with M1-specific markers CD80 and CD86, while positively correlated with M2-specific marker CD163 in macrophages, further supporting the conclusion that CMTM6 promotes M2 polarization. In addition, knockdown of CMTM6 in OSCC cells promoted the expression of pro-inflammatory factors TNF-α and IL-12p40 in macrophages and reduce the expression of anti-inflammatory factor IL-10 in macrophages. The authors further explored the mechanism by which OSCC cells influence M2 polarization. The results were surprising to find that CMTM6 can shuttle to macrophages through cell-derived exosomes and activate ERK1/2 signaling to promote M2 polarization (77). Other studies also found that CMTM6 expression was positively correlated with the M2 macrophage polarization-related genes such as IL-10, STAT3 and IL-33 in CRC, which further supporting the above viewpoint (28). Therefore, the polarizaiton of M2 macrophages may be regulated by CMTM6 (**Figure 1**).

### CMTM6 regulates the functions of MDSCs in TME

Tumor cells can remodel myeloid cells including neutrophils and monocytes, which makes them showing increased immature phenotype and morphology, decreased phagocytosis and enhanced secretion of anti-inflammatory cytokines (78). These immature myeloid cells consequently proliferate and are converted to myeloid-derived suppressor cells  (79). MDSCs, an important class of immunosuppressive cells in the TME, exert the immunosuppressive function mainly by enhancing the secretion of IL-10 and TGF-β and promoting the expression of PD-L1 (79). Wang et al. analyzed the tumor microenvironment in BALB/c mice models implanted with CMTM6 knockdown Renca cells and found that knockdown of CMTM6 in tumors can significantly reduce the infiltration MDSCs in TME (27).

### CMTM6 regulates the functions of Tregs in TME

Tregs are essential for mainting immune tolerance by suppressing host immunity to self and nonself antigens (80). Tregs can be recruited into tumor cells by chemokines in TME (81). Tregs are infiltrated into tumors, and secret inhibitory cytokines to exert their suppressive function by limiting the activation and proliferation of effector T cells and NK cells (82, 83). Through analyzing GSVA data, Guan et al. found that the differentiation of Treg was positively correlated with the expression of CMTM6, which implies that CMTM6 can promote the immune escape of glioma by inhibiting the function of T cells (20).

### CMTM6 regulates the functions of DCs in TME

DCs are major antigen-presenting cells involved in the regulating anticancer immunity (84). The mature DCs capture tumor-associated antigens (TAAs) by binding with their MHC molecules and deliver TAAs to T cells, subsequently initiate and activate the effector T cells (85). The mature DCs also secrete immunostimulatory cytokines to activate T cells against tumors (85). The TME induces the population of immature DCs or the DCs without function of activating T cells, which may even lead to immune tolerance and then impair antitumor immunity (84). Mezzadra et al. reported that CMTM6 is not only expressed on tumor cells, but also on DCs (13). CIBERSORT method was used to evaluate differences in the composition of immune cells between CMTM6 high and CMTM6 low groups of lung adenocarcinoma samples (68). DCs were included in the 11 immune cells affected by CMTM6 expression. The proportion of DCs was significantly positively correlated with the expression of CMTM6 (68). Yin et al. investigated the correlation between CMTM6 and immune cells in ovarian cancer by means of bioinformatics analysis (22). The infiltration of dendritic cells was closely associated with CMTM6 expression in ovarian cancer (22). Therefore, CMTM6 might has a certain regulatory effect on DC.

### CMTM6 and CMTM4 regulate the functions of CD8^+^ T cells in TME

T cell receptor (TCR) interacts with the tumors or DCs` MHC class I molecules bound to TAA, which turns naive CD8^+^ T cells into cytotoxic T lymphocytes (CTLs). CTLs are transported and infiltrated into tumors through blood or lymphatic vessels (79). CTLs can directly kill tumor cells by releasing perforin, granzyme, interferon-γ, and TNF-α. However, in TME, CTLs will encounter an immunosuppressive environment and they will enter the “exhaustion” state with decreased proliferation and reduced production of cytotoxic mediators. The immunosuppressive mediators released by tumor and stromal cells in the TME negatively regulate CTLs-mediated tumor killing by inducing expression of indoleamine 2,3-dioxygenase 1, PD-L1, cyclooxygenase 2, and STAT3 (79, 86).

CMTM6 promotes tumor progression in multiple tumor types through PD-L1-mediated T cells suppressing (18, 20). Depletion of CMTM6 in melanoma significantly reduces PD-L1 expression and promotes CD8^+^ T cell activity (12, 13). Burr et al. found that CTLs can more effectively kill melanoma cells with reduced PD-L1 expression following CMTM6 knockout (12). The activity of CTLs could be enhanced when co-cultured with CMTM6 depletion tumor cells, mainly manifested by increased secretion of perforin, TNF-α, IFN-γ and IL-2. CMTM6 has also been validated to inhibit T cell activation and antitumor responses in mouse melanoma models. Similarly, Mezzadra et al. concluded that CMTM6 depletion ameliorates PD-L1-mediated T cell suppression (**Figure 1**) (13).

Accumulating evidences indicated that CMTM6 may play a significant role in T cell suppression in many tumors. Guan et al. found that CMTM6 may inhibit the antitumor immunity of T cells in glioma through the positive regulation of PD-L1 (20). Chen et al. found that the expression levels of immune checkpoint markers such as B7-H3, LAG-3, VISTA, TIM-3 and PD-L1 in HNSCC were highly positively correlated with CMTM6 protein levels (18). Their results indicated that CMTM6 protein may promote tumor immune escape in HNSCC by inhibiting the function of effector T cells (18). In their xenograft C3H/He mice models, depletion of CMTM6 significantly increased the infiltrating proportion of T cells (CD4^+^ and CD8^+^ T cells) in tumor. ELISA results additionally showed that the expression of CMTM6 was negatively correlated with the secretion of INF-γ, TNF-α and granzyme B, which suggests that T lymphocyte was activated upon CMTM6 knockout *in vivo*. Wang et al. analyzed the alteration of TME in CMTM6 knockdown renal cell carcinomas of xenograft mouse models and found that T cells (CD4^+^ and CD8^+^) were significantly increased in the shCMTM6 group (27).

CMTM4 has the same function as CMTM6 to stabilize PD-L1, therefore, it may also negatively regulate CD8^+^ T cells and suppress T cell antitumor immunity. Routh et al. found that CMTM4 was enriched in low CD8^+^ T cell infiltration tumors by analyzing RNA-seq data from 23 solid tumor sources (87). In HCC, CMTM4 mRNA levels had negative correlations with cytotoxic cells which indicated that CMTM4 negatively influenced immune cell infiltration in HCC tissues (70). Chui et al. found that CMTM4 could promote HCC growth in immunocompetent mice by inhibiting the infiltration of CD8^+^ T cells (88) **(** **Figure 1** **)**.

### CMTM6 and CMTM4 regulate the functions of immune cells through stabilizing PD-L1 protein

It was reported that CMTM6 and CMTM4 are directly and/or indirectly regulates the expression of PD-L1 in cancer cells and in different immune cells including DCs, macrophages, and monocytes (13, 28, 33, 77). Furthermore, CMTM6 expression was positively correlated with PD-L1 expression in various cancers including HNSCC (18), lung cancer (30, 69), gliomas (20), gastric cancer (33, 36), and colon cancer (40) (**Table 1**). Similarly, CMTM4 was also identified as the positive regulator of PD-L1 in many tumors, such as in HCC (88) and in HNSCC (42). Mechanistically, CMTM6 could stabilize PD-L1 by preventing PD-L1 from lysosome-mediated degradation. CMTM6 depletion does not affect MHC-I expression but reduces PD-L1 expression (12). In line with the notion, Mezzadra et al. identified the function of stabilization of PD-L1 protein by CMTM6, which is shared by CMTM4 but not other CMTM family members (13). Therefore, PD-L1 might depend on CMTM6/4 to efficiently exert its immunosuppressive function. It is well known that the PD-1/PD-L1 pathway is the significant mechanism of tumor immune escape (89, 90). PD-1 is an important co-inhibitory receptor on T cells. Upon PD-1 binding to its ligand, Src homology 2 domain-containing protein tyrosine phosphatase-2 will be recruited after phosphorylation of PD-1. This event will lead to limit the function of effector T cells by inhibiting the TCR and CD28 pathways (91, 92) (**Figure 1**).

### Exosome mediated intercellular shuttling of CMTM6 in TME

Extracellular vesicles (EVs) are mainly divided into exosomes, microvesicles and apoptotic bodies. Most cells could secrete EVs both under physiological and cellular stress conditions (93). Recent evidences suggest that EVs, particularly exosomes, can regulate interaction between cells in TME (94–96). Several studies have reported that EVs induce immune tolerance in TME, contributing to the immunosuppressive effect (97, 98). EVs secreted by tumor cells can carry immunosuppressive molecules including PD-L1, Fas ligand (Fas-L), TGF-β and prostaglandin E2 to the surrounding immune cells and contribute to the occurrence and development of tumors (99–104). Pang et al. have found that CMTM6 regulates the physiological state of immune cells in TME through exosome mediated shuttling (77). CMTM6 was found in M0 macrophages co-cultured with OSCC cells and contained in the exosomes derived from OSCC cells. It suggested that CMTM6 can shuttle to macrophages from OSCC through exosomes secreted by OSCC cells (77). Therefore, exosomal CMTM6can promote the polarization of M2-like macrophages which contributes to an immunosuppressive state of TME (**Figure 1**).

### CMTM6 and CMTM4 affect proliferation, migration, and invasion of tumor cell in TME

**Figure 2:**
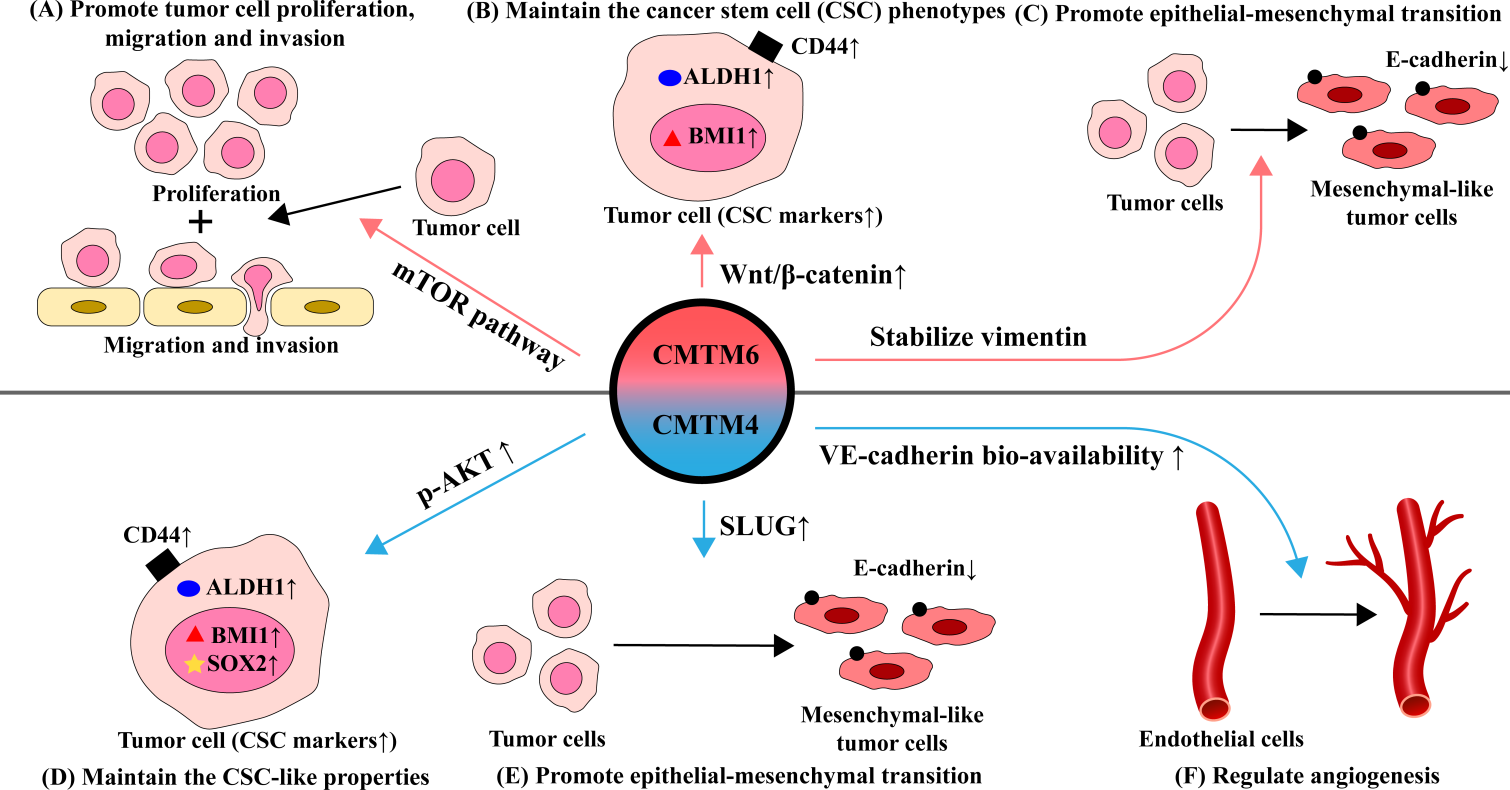
Effects and mechanisms of CMTM6 and CMTM4 in regulating the function of tumor and endothelial cells in TME. **(A)** CMTM6 promotes tumor proliferation, migration and invasion by activating the mTOR pathway. **(B)** CMTM6 maintains stem cell-like properties through affect Wnt/β-catenin signaling pathway, mainly manifested by elevated expression of several CSC-related markers such as CD44, ALDH and BMI1. **(C)** CMTM6 induces epithelial-mesenchymal transition (EMT) by stabilizing vimentin, which behaved as the E-cadherin decreased. **(D)** CMTM4 positively affected CSC-like properties *via* the AKT pathway, mainly manifested by significantly elevated expression of CSC markers (CD44, ALDH1, BMI1, and SOX2). **(E)** CMTM4 promotes EMT through positive regulation of SLUG. **(F)** CMTM4 plays an important role in regulating angiogenesis by enhancing the bio-availability of VE-cadherin at cell-cell adherens junctions and promoting endothelial barrier function.

CMTM6 and CMTM4 can affect tumor proliferation, metastasis, maintain the tumor stem cells phenotypes and promote epithelial-mesenchymal transition (EMT). CMTM6 promoted tumor proliferation, migration, and invasion in various tumors, such as RCC (27), gliomas (19), and HCC (105). Wei et al. found that in gliomas, depletion of CMTM6 inactivated the mTOR pathway and exerted a suppressive function on glioma cell behaviors (the proliferation, invasion, and migration) (19) (**Figure 2**). Moreover, CMTM4 was identified as one of the 37 genes needed for cell division (106). Li et al. found that CMTM4 knockdown by small interfering RNA inhibited the migration and invasion abilities of HNSCC cells (42). However, CMTM4 has also been reported to play a tumor suppressor role in some tumors, which can inhibit cell proliferation in clear cell renal cell carcinoma (107) and colorectal cancer (108).

### CMTM6 and CMTM4 maintain the cancer stem cell phenotypes

Cancer stem cells (CSCs) are cells with stem cell properties in tumor tissues. They have both multicellular differentiation potential and self-renewal ability. CSCs reprogram relevant cells in the tumor microenvironment to favor tumor development while suppressing the tumor-killing effect of immune cells (109, 110). Chen et al. found that targeting CMTM6 suppresses stem cell-like properties in HNSCC (18). Elevation of CMTM6 contributes to maintaining the CSC phenotype in HNSCC. After CMTM6 depletion, the expression of several CSC-related markers including ALDH1, CD44, and BMI1 were significantly downregulated. Wnt/β-catenin signaling is essential for cancer cell stemness and differentiation with mesenchymal features (111, 112). Interestingly, CMTM6 is highly associated with the Wnt/β-catenin signaling pathway and CMTM6 may influence the maintenance of CSC through this pathway (18). CMTM6 was positively correlated with the expression and the nuclear translocation of β-catenin in HNSCC. It was also reported that knockdown of CMTM4 negatively affected CSC-like properties *via* the AKT pathway and was manifested by reduced expression of SOX2, CD44, BMI1 and ALDH1 (42) (**Figure 2**).

### CMTM6 and CMTM4 promote epithelial-mesenchymal transition

Epithelial-mesenchymal transition (EMT) is the process by which epithelial phenotype cells differentiate into mesenchymal phenotype cells. Epithelial cells gain the ability to migrate and invade while losing polarity and intercellular adhesion during EMT. EMT contributes to tumor proliferation, metastasis, tumor stem cell differentiation, and drug resistance. EMT-induced epithelial plasticity could be manifested by changes in the expression of epithelial markers including E-cadherin and mesenchymal proteins including N-cadherin and vimentin (113). Recent study demonstrated that CMTM6 induces EMT by stabilizing vimentin, which in turn promote migration and invasion of tumor cells in HCC (105). The effect of CMTM6 on EMT may be mediated through Wnt/β-catenin signaling (18). Li et al. found that the EMT process was downregulated by knockdown of CMTM4 (42). Mechanically, CMTM4 was positively correlated with SLUG which is a pivotal associated transcription factor mediating the activation of EMT. Furthermore, CMTM4 inhibited the expression of E-cadherin and conversely promotesd the expression of MMP2, SNAIL and N-cadherin expression (**Figure 2**).

### CMTM4 regulates angiogenesis through modulating the functions of endothelial cells

Chrifi et al. have identified CMTM4 silencing impaired vascular sprouting and growth *in vitro* and *in vivo* and discovered a key role for CMTM4 in regulating angiogenesis (114). Mechanistically, VE-cadherin can co-localize with CMTM4. CMTM4 promotes the recycling of the VE-cadherin to the cell surface of endothelial adherens junctions, which enhances the endothelial barrier function and improves the bio-availability of VE-cadherin. However, the underlying mechanisms for CMTM4 in regulating angiogenesis in cancer needs further in deep investigation (**Figure 2**).

### Potential clinical application of CMTM6 and CMTM4

Currently, immunotherapy has achieved great success in cancer treatments and lead a new direction in tumor therapy. However, the response rates to immunotherapy vary among tumor types due to the complex TME (115–117). Identification of new and effective immunotherapy targets in TME might help to maximize the clinical efficacy of immune checkpoint inhibition-resistant patients (17). To improve the efficacy of immunotherapy and predict patient outcomes, better efficacy and safety biomarkers need to be discovered to better describe TME (118, 119). CMTM6 and CMTM4 might have a high potential for clinical application, including as biomarkers to predict efficacy and as new or combined immunotherapy targets to enhance the clinical benefits of immunotherapy.

CMTM6/4 can stabilize PD-L1 protein, promote the polarization of M2 macrophages and negatively regulate CD8^+^ T cells activity, which tends to build an immunosuppressive TME and contribute to tumor immune escape. Chui et al. found that CMTM4 depletion sensitized HCC tumors to anti-PD-L1 treatment (88). Therefore, CMTM6 and CMTM4 may be attractive to development novel immunotherapeutic stategy for cancer patients with poor outcomes treated by current methods.

Currently, many biomarkers demonstrate the capability to effectively predict antitumor response, such as PD-L1 expression, mismatch repair deficiency, tumor mutational burden, tumor neoantigen burden, and TILs (120–123). However, these markers may not fully explain the underlying mechanisms for the lack of response to checkpoint blockade observed in the majority of patients (124–127). In many tumors, the CMTM6 and CMTM4 expression levels correlate with patient response to PD-1/PD-L1 blockade (28, 30, 31, 38, 88). Patients with high expression of CMTM6 in macrophages can obtain the greatest benefit from PD-1/PD-L1 blockade in colorectal cancer (28) and in NSCLC (41). Wang et al. found that Co-expression of CMTM6 or CMTM4 with PD-L1 on tumor cells can predict the therapeutic efficacy of anti-PD-1/L1 in gastric adenocarcinoma (33). Another study showed that high coexpression of CMTM6 and PD-L1 in stromal compartment was significantly associated with longer survival in treated patients (38). Recent evidence indicated that tumor-derived EVs can be a potential cancer immunotherapy biomarker which speculating that exosomal CMTM6 is a potential safety biomarker (77). If the expression levels of CMTM6 and CMTM4 are potential biomarkers to predict the efficacy of immunotherapy, then it will facilitate the development of novel predictive models for patient screening before immunotherapy and the personalized medicine. Furthermore, CMTM6 also confers resistance to cisplatin by regulating Wnt signaling through the ENO-1/AKT/GSK3β axis. Therefore, CMTM6 could be a new promising drug-resistant therapeutic target (128).

## Conclusion and outlook

CMTM6 and CMTM4 are novel proteins found to promote tumor progression by stabilizing PD-L1 in recent and ongoing research. The expression of CMTM6 and CMTM4 correlated heavily with the prognosis of various cancers. CMTM6 has a tumor-promoting effect and CMTM6 is associated with a poor prognosis, especiallyin gastric cancer (34) and HNSCC (18). Additionally, CMTM6 and CMTM4 have been reported that they tend to establish an immunosuppressive microenvironment in tumors, such as gliomas, renal carcinomas and CRC (20, 27, 28). The relationship between CMTM6 and many kinds of cells in TME has been well studied. These cells included M2 macrophage, CTL, and MDSC (12, 27, 77). CMTM6 can shuttle to macrophages through tumor cell-derived exosomes, which induces M2-like macrophage polarization (77). However, currently there is a limited number of studies on CMTM4. Whether CMTM4 has the same function warrants further in deep investigation to better explore the clinical application of CMTM4.

Although CMTM6 plays an important role in the TME, the regulatory pathways for CMTM6/4 in regulating different components of TME are complicated and vary in different tumor microenvironments which implicated that the effect of CMTM6/4 on TME might be cancer type dependent. The overall landscape analysis of CMTM6 and CMTM4 in multiple tumors may be useful for understanding their function. It is very interesting whether CMTM6 and CMTM4 could be new targets to benefit immunotherapy which might warrant further in deep investigation to facilitate the immunotherapy.

Understanding the mechanisms underlying the regulation of CMTM6/4 might be helpful to develop new therapeutic method to target CMTM6/4. Liu et al. found HuR stabilizes CMTM6 mRNA *via* direct association with AU-rich elements in its 3′UTR and predominantly upregulates CMTM6 (129). Furthermore, CMTM6 expression levels in OV are also regulated by copy number variation CNVs and epigenetic modifications of DNA (22). CDR1-AS promotes PD-L1 expression in colorectal cancer by enhancing CMTM6/4 expression (40). Moreover, The expression of CMTM6 and its interaction with PD-L1 are involved and regulated by ATM and WEE1 enzymes (130). Recent studies suggest that NRP1 may be involved in the maintenance of CMTM6 stability (24). There is still a lack of relevant research on CMTM4.

In summary, CMTM6 and CMTM4 play important roles in the TME through regulating PD-L1, immune cells, tumor cells and different components of TME. CMTM6 and CMTM4 might have great potential as biomarkers to predict immunotherapy efficacy and as targets to improve the immunotherapy efficacy in combination with PD-L1 blockage.

## Author contributions

TZ wrote the manuscript and drew the pictures with partial help from HY. XZ and XD edited and revised the manuscript. All authors approved the final manuscript.

## Fundings

This work was partly supported by the National Natural Science Foundation of China (No: 81972558), the “Startup funding of First Hospital, JLU”, the Natural Science Foundation of Jilin Province (No: 20200201367JC, No: 20210204165YY, No: 20200201473JC, No: 20210101245JC).

## Acknowledgments

We thank other members of the Zhang laboratory and Dai laboratory for critical reading of the manuscript and useful discussions.

## Conflict of interest

The authors declare that the research was conducted in the absence of any commercial or financial relationships that could be construed as a potential conflict of interest.

## Publisher’s note

All claims expressed in this article are solely those of the authors and do not necessarily represent those of their affiliated organizations, or those of the publisher, the editors and the reviewers. Any product that may be evaluated in this article, or claim that may be made by its manufacturer, is not guaranteed or endorsed by the publisher.
